# KDELR1 Is an Independent Prognostic Predictor and Correlates With Immunity in Glioma

**DOI:** 10.3389/fonc.2022.783721

**Published:** 2022-06-23

**Authors:** Yifan Yuan, Biao Yang, Zengxin Qi, Zhenyuan Han, Jiajun Cai, Jianping Song

**Affiliations:** ^1^Department of Neurosurgery, Huashan Hospital, Fudan University, Shanghai, China; ^2^National Center for Neurological Disorders, Shanghai, China; ^3^Shanghai Key Laboratory of Brain Function Restoration and Neural Regeneration, Shanghai, China; ^4^State Key Laboratory of Medical Neurobiology and MOE Frontiers Center for Brain Science, School of Basic Medical Sciences and Institutes of Brain Science, Fudan University, Shanghai, China; ^5^Department of Oral and Maxillofacial Surgery, Peking University School and Hospital of Stomatology, Beijing, China; ^6^Neurosurgical Institute of Fudan University, Shanghai, China; ^7^Shanghai Clinical Medical Center of Neurosurgery, Shanghai, China

**Keywords:** KDELR1, biomarker, immunity, prognosis, glioma

## Abstract

**Background:**

Gliomas are the most malignant central nervous system tumors. With the development of sequencing technology, more potential biomarkers related to the treatment, prognosis, and molecular classification of glioma have been identified. Here, we intend to investigate the potential biological function and clinical value of a new biomarker in glioma.

**Methods:**

KDELR1 expression data and the corresponding clinical information were downloaded from public databases and then preprocessed using R language. Correlation, Kaplan–Meier survival, and Cox regression analyses were performed to explore the clinical significance of KDELR1 in glioma patients. Furthermore, the immune infiltration and microenvironment parameters were evaluated *via* TIMER and CIBERSORT. Immunohistochemistry was conducted to confirm the KDELR1 expression and its correlation with immunity infiltration and prognosis.

**Results:**

KDELR1 was upregulated in glioma samples compared with normal brain tissues, and its expression was significantly correlated with age, the World Health Organization (WHO) grade, recurrence, necrosis, microvascular proliferation, molecular classification, isocitrate dehydrogenase (IDH) mutation, and 1p/19q codeletion status. In addition, survival analysis showed that glioma patients with KDELR1 overexpression had shorter overall survival (OS) and disease-free survival times, and Cox regression analysis revealed that KDELR1 acted as an independent prognostic factor of OS in glioma patients. Gene set enrichment analysis indicated a significant enrichment of metabolism-associated pathways. KDELR1 expression was positively associated with immune infiltration (including infiltration by CD8+ T cells, CD4+ T cells, macrophages, and so on) and microenvironment parameters (including stromal, immune, and ESTIMATE scores) in gliomas. The expression of KDELR1 and its correlation with the tumor grade and prognosis were confirmed by immunohistochemistry in clinical samples (n = 119, P < 0.05).

**Conclusions:**

Taken together, these findings suggest that KDELR1 is correlated with the tumor grade, molecular classifications, and immune infiltration; highlighting that KDELR1 is a novel and promising biomarker for molecular classification, treatment, and prognostic assessment may further indicate the treating effect of immune therapy.

## Introduction

Gliomas are the most common primary malignant tumors of the central nervous system (CNS), accounting for approximately 40%~60% of intracranial tumors, and have high morbidity and mortality (1). Low-grade gliomas (LGGs) and high-grade gliomas (HGGs) describe grade I/II and grade III/IV gliomas, respectively. Grade IV glioma, or glioblastoma multiforme (GBM), has a poor prognosis, with a median survival time of only 14–16 months from the first diagnosis (2, 3). Currently, new strategies like precise surgery, adjuvant radiotherapy, and chemotherapy (4, 5) have been conducted, while the prognosis of glioma has not improved significantly to date, and the recurrence rate is still high (6).

Compared with the World Health Organization (WHO) classification of CNS tumors published in 2007 and 2016, the main change in the 2021 classification is laying more emphasis on genetic parameters into the strategy of glioma diagnosis, breaking with the century-old standard of diagnosis based entirely on microscopy (3, 7) . The Cancer Genome Atlas (TCGA) team has subclassified GBMs into four subtypes: proneural, mesenchymal, neural, and classical (8). In addition, Phillips et al. divided HGGs into proneural, mesenchymal, and proliferative subtypes (9). Notably, patients with mesenchymal subtype glioma usually have a worse prognosis than patients with the proneural subtype (10). In addition, mutations in the telomerase reverse transcriptase (TERT) promoter and IDH were found to be beneficial for the classification and risk stratification of grade II/III glioma patients (11, 12).

Given the limited effectiveness of traditional treatments, a series of new treating strategies have been tried in gliomas with the help of advancing genomics. In recent years, high hopes have been placed on tumor immunotherapy, which has not only gradually become a research hotspot in the scientific studies (13) but also achieved amazing curative effects in the clinical treatment of some patients. Unfortunately, only limited patients with gliomas prolong the survival time after immune treatments (14). At present, relevant basic studies have revealed that the expression (15) or combinations (16–18) of immune-related indicators can be used to predict the prognosis of GBM patients, which suggests that finding a molecular target that can effectively predict the immune status of patients is urgent for guiding treatment (19).

KDEL Endoplasmic Reticulum Protein Retention Receptor 1 (KDELR1), a 24.5-kDa seven-transmembrane protein encoded by ERD2, which is located on 19q13.33, is responsible for the retrieval of soluble endoplasmic reticulum (ER) residents from the Golgi back to the ER (20). Some studies reported that KDELR1 mainly regulates the retention of soluble ER residents and the transporting processes in the secretory pathway (21, 22). Some recent studies showed that KDELR1 might participate in maintaining cellular homeostasis (23) along with a potential role that involves the regulation of integrated stress responses (ISRs) in T cells (24), which is a possible way of regulating the immune homeostasis. However, the roles of KDELR1 in the biological functions and molecular mechanisms of gliomas are not clear.

In this study, genetic and clinical data related to KDELR1 were downloaded from public databases, including TCGA, Gene Expression Omnibus (GEO), and Chinese Glioma Genome Atlas (CGGA). After data preprocessing was completed, correlation, survival, and Cox regression analyses were performed to explore the potential function of KDELR1. We aimed to develop a novel diagnostic biomarker for glioma, thus assisting disease stratification and precise treatment.

## Materials and Methods

### Data Downloading and Preprocessing

The datasets of expression profiles with corresponding clinical information were obtained from public databases, including TCGA (https://cancergenome.nih.gov/), CGGA (http://www.cgga.org.cn/), and GEO (https://www.ncbi.nlm.nih.gov/geo/). The TCGA database classifies gliomas into two types: LGG and GBM; therefore, the three cohorts (glioma, LGG, and GBM) were named TCGA_glioma, TCGA_LGG, and TCGA_GBM, respectively. These three datasets contained 698, 529, and 169 samples, respectively. For Chinese cohorts, CGGA contained three datasets: mRNA-array_301, mRNAseq_325, and mRNAseq_693, each with 301, 325, and 693 glioma samples, respectively. The CGGA database contains abundant clinical information, such as age, sex, WHO grade, TCGA subtype, histology, IDH mutation and 1p/19q codeletion status, and OS time and status. In addition, microarray datasets were obtained from GEO, including GSE4271 (generated from GPL96), GSE4290 (GPL570), GSE4412 (GPL96), GSE68848 (GPL570), and GSE13041 (GPL96, GPL570, or GPL8300). After retrieval, all the gene expression profiles were preprocessed, including background correction, normalization, and log2 conversion, using R software (version 3.5.1). When a gene matched multiple probes, the average was computed and adopted for subsequent analyses.

To explore the expression levels of KDELR1 in brain or CNS cancer (especially gliomas) and normal samples, the KDELR1 gene was submitted to Oncomine (https://www.oncomine.org) with the criteria of P < 0.01, fold-change (FC) > 1.5 and gene rank = all (24, 25). In addition, Gene Expression Profiling Interactive Analysis 2 (GEPIA2; http://gepia.cancer-pku.cn/index.html), based on gene expression and clinical data from TCGA and GTEx, was used for the survival analyses of OS and disease-free survival (DFS) (26). Gene set enrichment analysis (GSEA) was used to analyze enrichment in high-risk and low-risk groups defined by KDELR1 expression levels (27).

### Analysis of Immune Infiltration and the Microenvironment

The R package “ESTIMATE” was used to analyze the communities of immune and stromal cells according to the characteristics of gene expression and then to obtain immune, stromal, and ESTIMATE scores (28). The significance of immune cells in the prognosis of glioma patients was explored in the Tumor Immune Estimation Resource (TIMER) site (29). TIMER was also used to provide an analysis of the clinical correlation between immune cell infiltration and patient survival.

### Clinical Specimen Collection and Immunochemistry Staining

Samples from 119 patients (5 patients with WHO grade I, 33 patients with WHO grade II, 28 patients with WHO grade III, and 53 patients with WHO grade IV glioma) were collected from Shanghai OutDo Biotech. Co., Ltd., Shanghai, China. Informed consent was obtained from all patients, and the experimental protocols were approved by the Ethics Committee of Shanghai OutDo Biotech Co., Ltd.

Antigen retrieval was performed by heating in the citrate buffer (10 mM, pH 6.0) for 10 min. The slides were incubated with the KDELR1 antibody (1:100, Cat# NBP2-12873-25 µg; Novus, Inc.), CD4 antibody (1: 200, Cat# ab 133616, Abcam, Inc.), and CD8 antibody (1:200, Cat# ab217344, Novus, Inc., Englewood, CO, US; Abcam, Inc.,Cambridge, UK; Agilent Technologies Inc., SantaClara, CA, US) for 1.5 h at room temperature. Immunoreactive elements were visualized using an EnVisio Detection kit (Cat# GK500705; Dako, Agilent Technologies, Inc.) containing the secondary antibody and peroxidase/3,3-diaminobenzidine (DAB) chromogen. Next, the cell nuclei were counterstained with hematoxylin. Slides in which the primary antibody was omitted were used as negative controls. KDELR1 immunoreactivity scores (IRS) were calculated based on the staining intensity (SP) and the positive staining percentage (SI) of the cells (the score was evaluated by two pathologists individually). SI was scored as follows: 0: <5%; 1: 5%–25%; 2: 25%–50%; 3: 51%–75% and 4: 75%–100%. SP was subjectively scored as follows: 0, no staining; 1, weak but definite staining; 2, moderate staining; and 3, intense staining. The IRS was calculated as IRS = SP + SI. The total possible score was 7, and specimens were assigned to one of the 4 levels based on the score: 0–1 (–), 2–3 (+), 4–5 (++), and more than 6 (+++).

### Statistical Analysis

GraphPad Prism 7.0, SPSS Statistics 20, and R language were used for statistical analysis. The box plots of the expression level of KDELR1 across different groups were generated and calculated by GraphPad Prism 7.0. Kaplan–Meier survival analysis and Cox regression analysis were performed and generated *via* the R language. Immunoreactivity scores were analyzed and generated *via* SPSS Statistics 20. Differences with P<0.05 were considered statistically significant.

## Results

### KDELR1 Expression Is Significantly Associated With Clinical Features and the Molecular Subtypes of Gliomas

**Figure 1:**
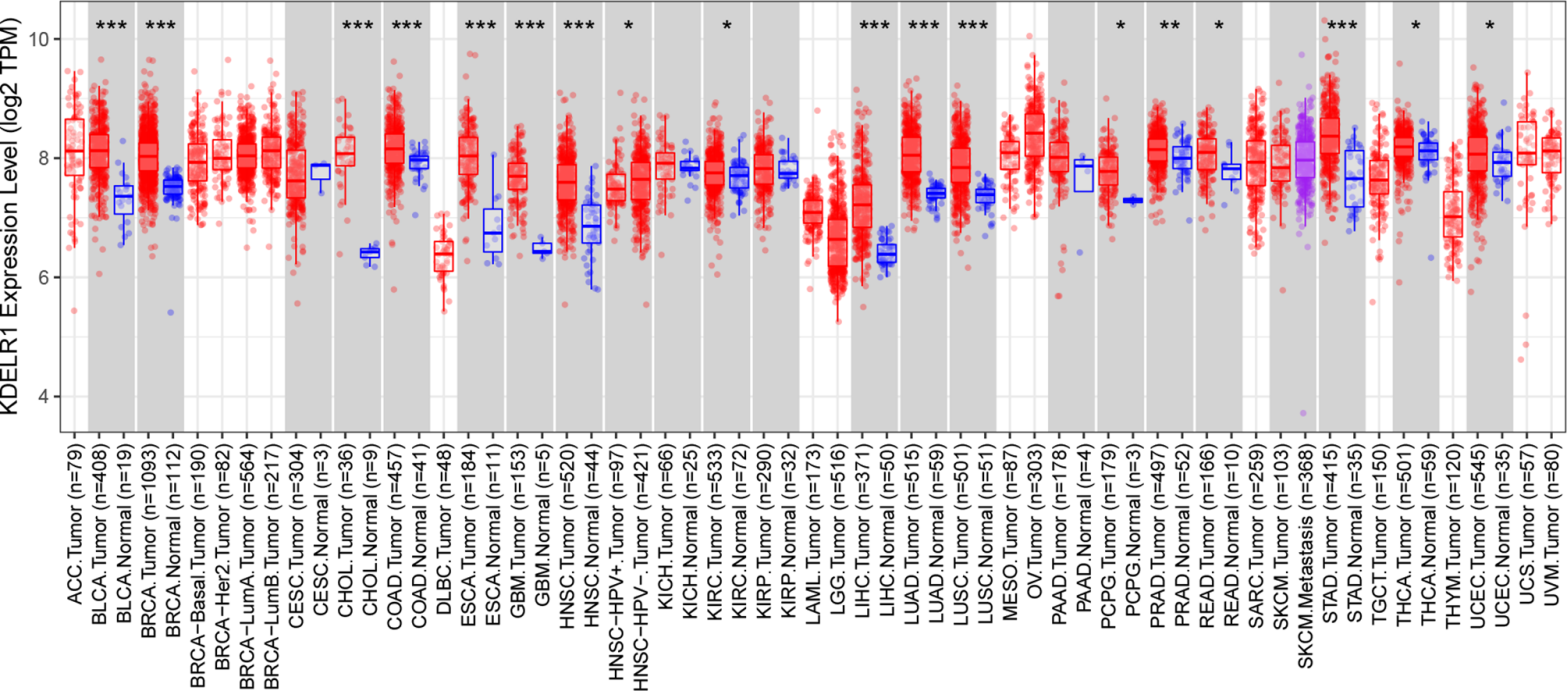
The expression level of KDELR1 is upregulated in GBM. The level of KDELR1 expression in different tumor types from TCGA data analyzed in TIMER. KDELR1 was highly expressed in bladder urothelial carcinoma (BLCA), breast invasive carcinoma (BRCA), cholangiocarcinoma (CHOL), colon adenocarcinoma (COAD), esophageal carcinoma (ESCA), glioblastoma (GBM), head and neck squamous cell carcinoma (HNSC), kidney renal clear cell carcinoma (KIRC), liver hepatocellular carcinoma (LIHC), lung adenocarcinoma (LUAD), lung squamous cell carcinoma (LUSC), pheochromocytoma and paraganglioma (PCPG), prostate adenocarcinoma (PRAD), rectum adenocarcinoma (READ), stomach adenocarcinoma (STAD), thyroid carcinoma (THCA), and uterine corpus endometrial carcinoma (UCEC) tissues and lowly expressed in the positive human papillomaviruses of head and neck squamous cell carcinoma (HNSC-HPV+). *P < 0.05, **P < 0.01, ***P < 0.001.

We initially evaluated KDELR1 transcription levels in different human tumors by analyzing TCGA RNA-seq data using the TIMER database (**Figure 1**). KDELR1 mRNA expression was markedly higher in GBM tissue than in normal brain tissue. In addition, KDELR1 was found to be highly expressed in bladder urothelial carcinoma (BLCA), breast-invasive carcinoma (BRCA), cholangiocarcinoma (CHOL), colon adenocarcinoma (COAD), esophageal carcinoma (ESCA), glioblastoma (GBM), head and neck squamous cell carcinoma (HNSC), kidney renal clear cell carcinoma (KIRC), liver hepatocellular carcinoma (LIHC), lung adenocarcinoma (LUAD), lung squamous cell carcinoma (LUSC), pheochromocytoma and paraganglioma (PCPG), prostate adenocarcinoma (PRAD), rectum adenocarcinoma (READ), stomach adenocarcinoma (STAD), thyroid carcinoma (THCA), and uterine corpus endometrial carcinoma (UCEC) tissues and was significantly lower in head and neck squamous cell carcinoma that were positive for human papillomaviruses (HNSC-HPV+) than in the respective control tissues. These results demonstrated that KDELR1 was abnormally expressed in multiple tumors. Overall, these results indicated that KDELR1 expression was higher in GBM tissues than in normal counterparts.

**Figure 2:**
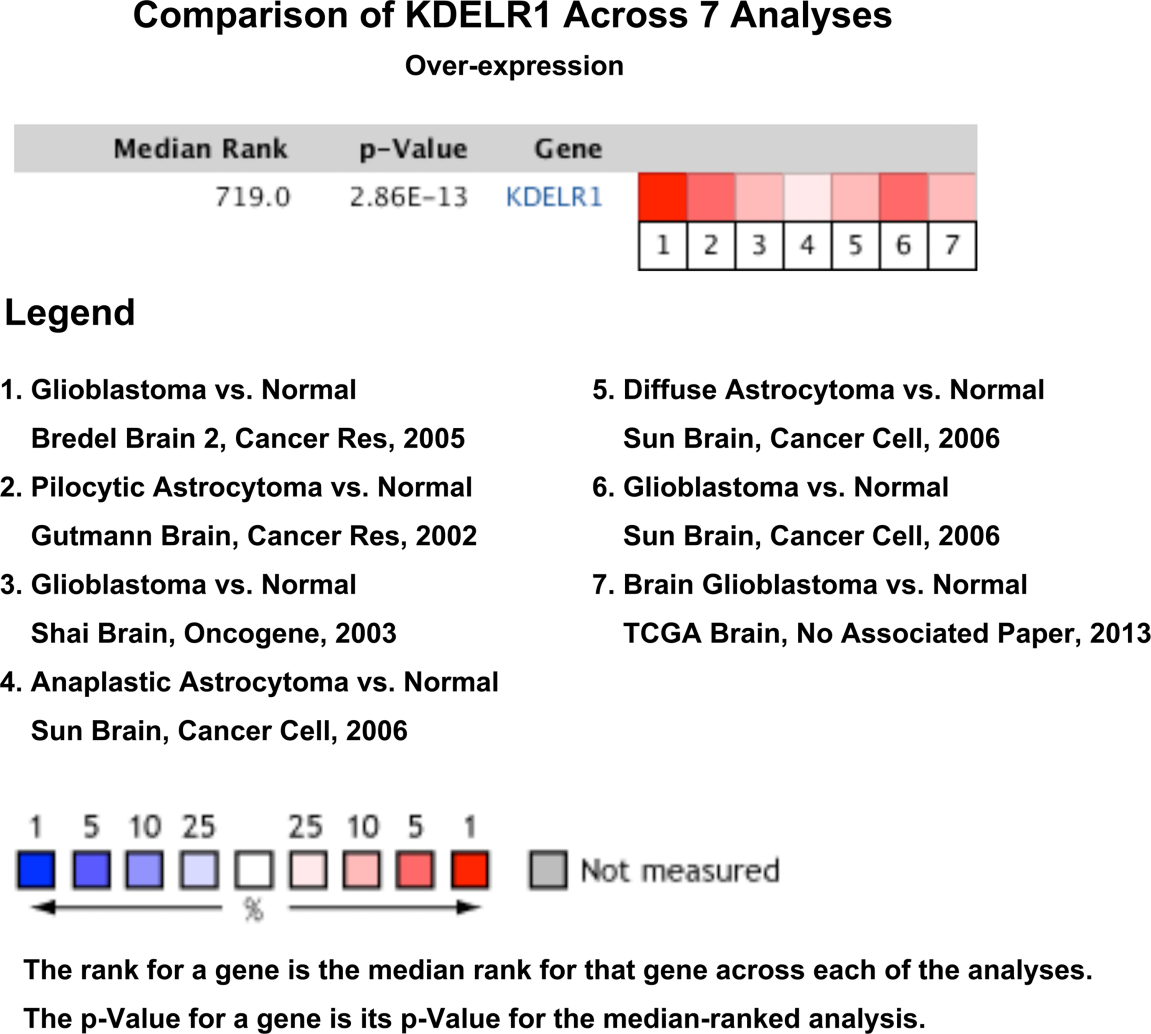
The expression levels of KDELR1 between brain or CNS cancer and corresponding normal samples with the criterion of P < 0.01, fold-change (FC) > 1.5 and gene rank = all using the database Oncomine.

The Oncomine search yielded seven analyses indicating KDELR1 upregulation and only one analysis indicating KDELR1 downregulation between brain or CNS cancer and normal samples with the criteria of P < 0.01, FC > 1.5, and gene rank = all (**Figure 2**). These results indicate that KDELR1 might be upregulated in CNS cancers, such as gliomas, compared with the corresponding normal samples.

### Overexpression of KDELR1 Is Positively Associated With Older Age, Recurrence, Necrosis, and Microvascular Proliferation in Gliomas

**Figure 3:**
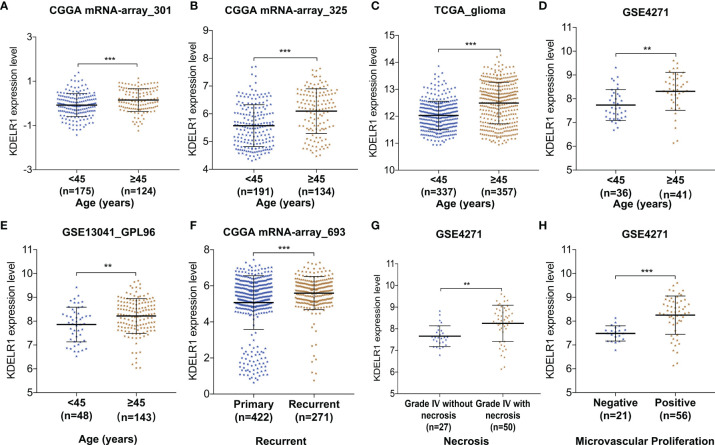
The relationships between KDELR1 expression and clinical features in glioma samples from different databases. **(A–E)** KDELR1 expression is significantly associated with age in mRNA-array_301 and mRNA-array_325 of CGGA, TCGA_glioma, GSE4271, and GSE13041 (GPL96), respectively. **(F)** The recurrent gliomas had a higher expression level of KDLER1 in CGGA mRNA-array_693. **(G)** The expression of KDLER1 is highly upregulated in grade IV gliomas with necrosis than ones without necrosis in the dataset GSE4271. **(H)** Gliomas with microvascular proliferation had a higher expression level of KDLER1 than gliomas without microvascular proliferation in GSE4271. **,P < 0.01; ***,P < 0.001.

To further clarify the relationship between KDELR1 expression and the clinical features of glioma patients, the glioma samples were classified into two or more groups according to each clinical feature in each dataset. Several datasets showed that KDELR1 expression was higher in glioma patients aged ≥45 years than in those aged <45 years, including mRNA-array_301 and mRNAseq_325 of CGGA, TGGA_glioma, GSE4271, and GSE13041 (GPL96) (P < 0.05; **Figures 3A–E**). In addition, recurrent glioma samples showed a higher expression level of KDELR1 than primary samples in the CGGA mRNA-array_693 dataset (P < 0.05; **Figure 3F**). Moreover, microvascular proliferation and necrosis are the diagnostic criteria of GBMs, and the analysis of the GSE4271 dataset showed that grade IV gliomas with necrosis or microvascular proliferation had much higher KDELR1 expression than those without necrosis (P < 0.05; **Figures 3G, H**).

### KDELR1 Expression Is Positively Related to the WHO Grade and Pathological Classification of Gliomas

**Figure 4:**
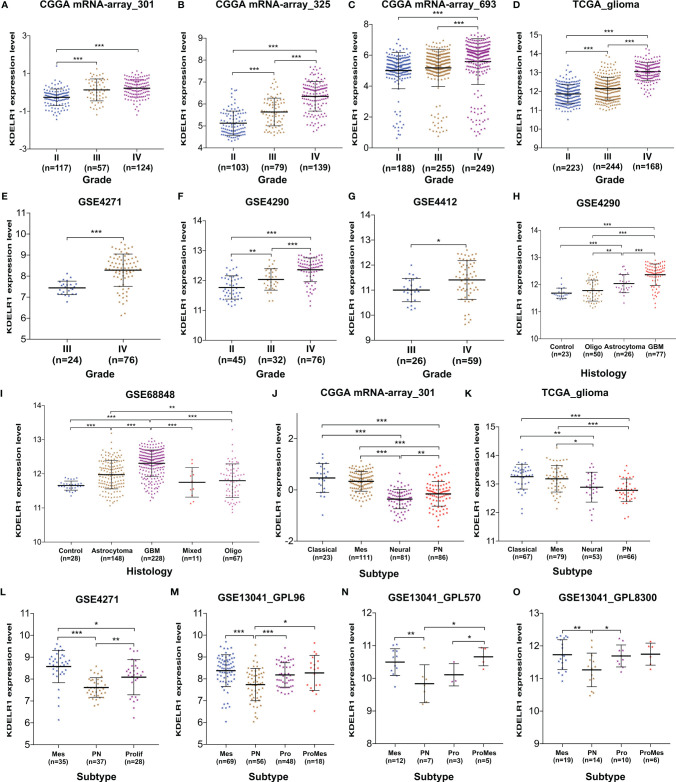
The associations between KDELR1 expression, and the WHO grades, histology, and molecular classification of gliomas. **(A–G)** KDELR1 expression significantly increased as the WHO grades of gliomas, including mRNA-array_301, mRNA-array_325, and mRNA-array_693 of CGGA, TCGA_glioma, GSE4271, GSE4290, and GSE4412. **(H)** Expression level of KDELR1 was gradually upregulated in the order of control, oligodendrogliomas, astrocytomas, and GBMs in GSE4290. **(I)** KDELR1 expression was upregulated in the order of control, oligodendrogliomas, astrocytomas, and GBMs in the dataset GSE68848. **(J–O)** Distribution of KDELR1 expression among distinct classification subtypes of gliomas in different datasets, including CGGA mRNA-array_301, TCGA_glioma, GSE4271, GSE4290, and GSE13041 (GPL96, GPL570, and GPL8300). *P < 0.05; **P < 0.01; ***P < 0.001.

Using the information from databases including GEO, CGGA, and TCGA, we wanted to determine whether the expression level of KDELR1 was related to the WHO grade and pathological classification of gliomas. The results showed that KDELR1 expression significantly increased with the WHO grade in several cohorts, including mRNA-array_301, mRNA-array_325, and mRNA-array_693 of CGGA, TCGA_glioma, GSE4271, GSE4290, and GSE4412 (P < 0.05; **Figures 4A–G**). Moreover, the expression level of KDELR1 was gradually upregulated along the sequence from control samples to oligodendrogliomas, astrocytomas, and GBMs in the GSE4290 dataset (P < 0.05; **Figure 4H**). Similarly, KDELR1 expression was upregulated from control samples to oligodendrogliomas, astrocytomas, and GBMs in the GSE68848 dataset (P < 0.05; **Figure 4I**). Taken together, these results indicate that KDELR1 expression is significantly related to the clinical features of gliomas and plays an important positive role in glioma progression.

### KDELR1 Expression Is Strongly Correlated With Molecular Classification and Biomarkers in Gliomas

With new developments in sequencing technology, more biomarkers of important clinical value have been identified and applied to clinical practices such as molecular classification, which improves risk stratification and treatment accuracy. To date, several similar molecular classifications of gliomas have been proposed by different research teams; the relationships between their corresponding subtypes and KDELR1 expression were analyzed in this study. The results from both CGGA mRNA-array_301 and TCGA_glioma showed that KDELR1 was frequently highly expressed in the classical and mesenchymal subtypes and weakly expressed in the proneural and neural subtypes of GBM (P < 0.05; **Figures 4J, K**). Similarly, mesenchymal-subtype gliomas had a significantly higher expression level of KDELR1 than proneural-subtype gliomas (P < 0.05; **Figures 4L–O**).

**Figure 5:**
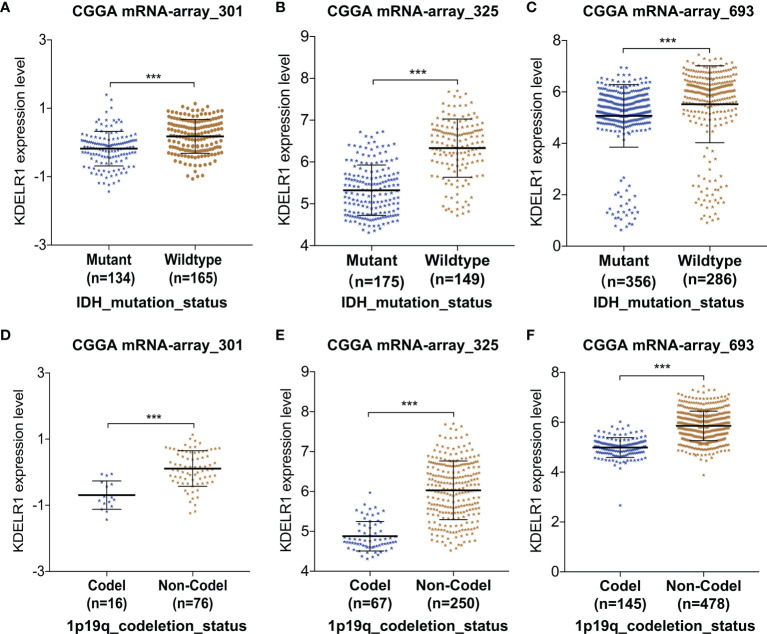
The relationships between KDELR1 expression and IDH mutation and the 1p/19q codeletion status in the database CGGA. **(A–C)** Gliomas with IDH mutation had a lower KDELR1 expression than gliomas with IDH wildtype. **(D–F)** Overexpression of KDELR1 was frequent to occur in the glioma samples without 1p/19q codeletion. ***P < 0.001.

Further, KDELR1 expression was found to be strongly correlated with molecular biomarkers such as IDH mutation and the 1p/19q codeletion status in the three datasets from CGGA (P < 0.05; **Figure 5**). The results showed that glioma patients with IDH mutation had lower KDELR1 expression than gliomas with IDH wildtype (P < 0.05; **Figures 5A–C**). KDELR1 overexpression frequently occurred in glioma samples without 1p/19q codeletion (P < 0.05; **Figures 5D–F**).

**Figure 6:**
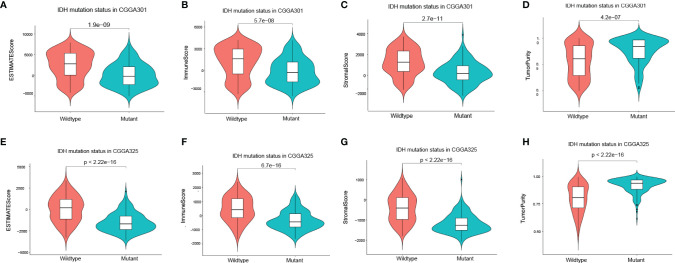
Correlation of the IDH mutation status with the tumor environment. **(A–H)** Violin plot showed the four differential scores (EstimateScore, ImmuneScore, StromalScore, TumorPurity) between IDH wildtype and mutation glioma samples by using the ESTIMATE algorithm in CGGA301 and CGGA325 datasets, respectively.

EstimateScore, ImmuneScore, StromalScore, and TumorPurity were analyzed between the two groups in CGGA301 and CGGA325 through the ESTIMATE algorithm (**Figures 6A–H**). ImmuneScore in IDH mutation samples was higher than in IDH-wildtype samples (**Figures 6B, F**). In summary, KDELR1 is strongly related to certain molecular biomarkers and the glioma classification, which indicates that KDELR1 could play a critical role in the development and molecular classification of gliomas.

### KDELR1 Acts as a Poor Prognostic Factor in Glioma Patients

**Figure 7:**
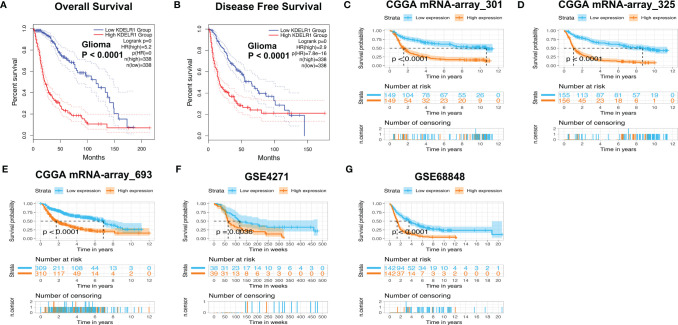
Survival analyses of KDELR1 in different datasets. **(A, B)** The low-KDELR1-expression group had a shorter overall survival (OS) and disease-free survival (DFS) time than the high-KDELR1-expression group using the online tool GEPIA2, respectively. **(C–G)** The low-KDELR1-expression group had a shorter OS time than the high-KDELR1-expression group using R language in the different datasets, including mRNA-array_301, mRNA-array_325, and mRNA-array_693 of CGGA, GSE4271, and GSE68848, respectively.

The above results showed that KDELR1 overexpression is positively associated with unfavorable clinical features, such as higher WHO grades, the mesenchymal subtype, recurrence, older age, and GBM, which indicates that KDELR1 might be an unfavorable prognostic factor in gliomas. When KDELR1 was submitted to the online tool GEPIA2, survival analyses indicated that glioma samples with KDELR1 overexpression had shorter OS and PFS (progression-free survival) times than those with low KDELR1 expression (P < 0.05; **Figures 7A, B**). Similarly, survival analyses revealed that the high-KDELR1-expression group had a shorter OS time than the low-KDELR1-expression group in six different datasets, including mRNA-array_301, mRNA-array_325, and mRNA-array_693 of CGGA, GSE4271, and GSE68848 (P < 0.05; **Figures 7C–G**).

### Cox Regression Analysis of KDELR1 as an Independent Predictor of Survival in Glioma Patients

As KDELR1 was associated with the prognosis of glioma patients, we wanted to determine whether it was an independent predictor of OS in gliomas using univariate and multivariate survival analyses. In the CGGA mRNA-array_301 dataset, univariate analysis showed that KDELR1 expression as well as age, the WHO grade, primary/recurrent/secondary type, histology, TCGA subtype, radiotherapy, chemotherapy, IDH mutation, and 1p/19q codeletion status were significantly associated with OS, and further multivariate analysis showed that KDELR1 expression and the chemotherapy status were independent prognostic predicators of OS in gliomas (**Table 1**). Moreover, similar results in the CCGA mRNA-array_325 and TCGA_glioma datasets showed that KDELR1 expression acts as an independent prognostic predicator in gliomas (**Tables 2**, **3**). Collectively, Cox regression analyses showed that KDELR1 is an independent prognostic predictor in glioma.

**Table 1:** Cox regression analysis of KDELR1 expression as an independent survival predictor of gliomas in CGGA mRNA-array_301.

Parameter	Univariate Analysis			Multivariate Analysis		
	P	HR	95%CI	P	HR	95%CI
Age	**P<0.001**	1.04	1.03–1.06	0.723	1.01	0.97–1.04
Gender	0.163	1.24	0.92–1.67	NA	NA	NA
WHO grade	**P<0.001**	2.70	2.23–3.26	0.130	1.37	0.91–2.07
PRS_type	**P<0.001**	2.21	1.67–2.94	0.758	1.17	0.44–3.09
Histology	**P<0.001**	1.13	1.08–1.19	0.343	1.10	0.90–1.34
TCGA_subtypes	**P<0.001**	0.62	0.53–0.72	0.186	1.28	0.89–1.86
Radio_status	**0.012**	0.58	0.37–0.89	**0.036**	0.44	0.21–0.95
Chemo_status	**0.021**	1.43	1.05–1.94	0.244	0.66	0.32–1.33
IDH_mutation_status	**P<0.001**	0.38	0.28–0.52	0.630	0.81	0.34–1.91
1p19q_codeletion_status	**P<0.001**	0.13	0.04–0.40	0.090	0.32	0.09–1.19
KDELR1 expression	**P<0.001**	2.80	2.11–3.71	**0.008**	3.00	1.33–6.77

CGGA, the Chinese Glioma Genome Altas; WHO, World Health Organization; A, astrocytomas; AA, anaplastic astrocytomas; AO, anaplastic oligodendrogliomas; AOA, anaplastic oligoastrocytomas; GBM, glioblastoma multiforme; O, oligodendrogliomas; OA, oligoastrocytomas; rA, recurrent astrocytomas; rAA, recurrent anaplastic astrocytomas; rAO, recurrent anaplastic oligodendrogliomas; rAOA, recurrent anaplastic oligoastrocytomas; rGBM, recurrent glioblastoma multiforme; sGBM, secondary glioblastoma multiforme; TCGA, The Cancer Genome Atlas; NA, not analyzed.

bold value: p value was less than 0.05 and the results were statistically significant.

**Table 2:** Cox regression analysis of KDELR1 expression as an independent survival predictor of gliomas in CGGA mRNAseq_325.

Parameter	Univariate Analysis			Multivariate Analysis		
	P	HR	95%CI	P	HR	95%CI
Age	**P<0.001**	1.03	1.02–1.04	**0.023**	1.02	1.00–1.03
Gender	0.613	0.93	0.71–1.23	NA	NA	NA
WHO grade	**P<0.001**	2.74	2.28–3.30	**P<0.001**	1.92	1.47–2.51
PRS_type	**P<0.001**	2.12	1.75–2.57	**0.016**	1.95	1.13–3.35
Histology	**P<0.001**	1.12	1.08–1.16	0.597	0.97	0.88–1.08
Radio_status	**P<0.001**	0.52	0.36–0.74	0.168	0.75	0.51–1.13
Chemo_status	**0.004**	1.55	1.15–2.1	**0.031**	0.68	0.49–0.97
IDH_mutation_status	**P<0.001**	0.38	0.29–0.51	0.912	0.98	0.66–1.46
1p19q_codeletion_status	**P<0.001**	0.17	0.10–0.28	**P<0.001**	0.36	0.20–0.64
KDELR1 expression	**P<0.001**	2.43	2.06–2.87	**0.028**	1.33	1.03–1.71

CGGA, the Chinese Glioma Genome Altas; WHO, World Health Organization; A, astrocytomas; AA, anaplastic astrocytomas; AO, anaplastic oligodendrogliomas; AOA, anaplastic oligoastrocytomas; GBM, glioblastoma multiforme; O, oligodendrogliomas; OA, oligoastrocytomas; rA, recurrent astrocytomas; rAA, recurrent anaplastic astrocytomas; rAO, recurrent anaplastic oligodendrogliomas; rAOA, recurrent anaplastic oligoastrocytomas; rGBM, recurrent glioblastoma multiforme; rOA, recurrent oligoastrocytomas; sGBM, secondary glioblastoma multiforme; NA, not analyzed.

bold value: p value was less than 0.05 and the results were statistically significant.

**Table 3:** Cox regression analysis of KDELR1 expression as an independent survival predictor of gliomas in TCGA.

Parameter	Univariate Analysis			Multivariate Analysis		
	P	HR	95%CI	P	HR	95%CI
Age	**P<0.001**	1.06	1.05–1.07	**P<0.001**	1.05	1.03–1.07
Gender	**0.047**	1.28	1.00–1.64	0.287	1.28	0.81–2.04
Race	0.749	0.94	0.64–1.39	NA	NA	NA
WHO grade	**P<0.001**	4.50	3.69–5.49	**0.002**	2.42	1.37–4.28
Histology	**P<0.001**	0.82	0.77–0.88	0.067	0.87	0.76–1.01
TCGA_subtypes	0.688	0.97	0.83–1.13	NA	NA	NA
KPS	**0.044**	0.57	0.33–0.99	0.118	0.61	0.33–1.13
Treatment_or_therapy	**P<0.001**	1.99	1.45–2.73	0.862	0.95	0.51–1.75
KDELR1 expression	**P<0.001**	3.32	2.80–3.94	**0.009**	1.75	1.15–2.68

TCGA, The Cancer Genome Atlas; WHO, World Health Organization; GBM, glioblastoma multiforme; KPS, Karnofsky performance score; NA, not analyzed.

bold value: p value was less than 0.05 and the results were statistically significant.

### KDELR1 Is Correlated With Immune Infiltration and the Microenvironment In Glioma

**Figure 8:**
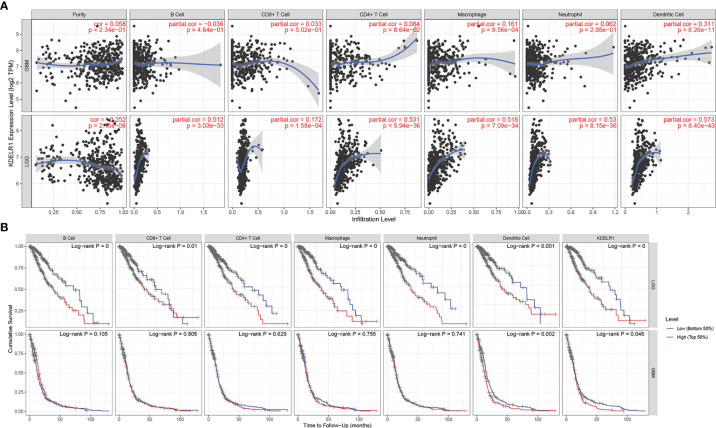
The relationship between the KDELR1 expression level, tumor purity, and immune cell infiltration was explored *via* the TIMER database. **(A)** KDELR1 was significantly correlated with immune cell infiltration in GBM and LGG patients. **(B)** Kaplan–Meier survival analysis of several immune cells in GBM and LGG patients.

**Figure 9:**
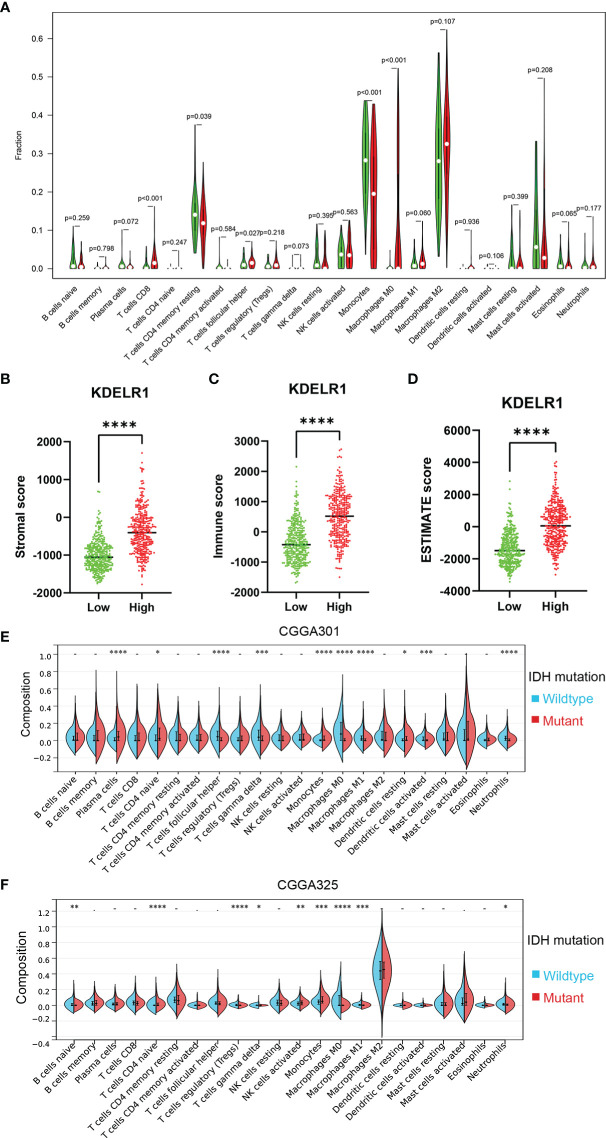
Relationship between the ESTIMATE score and the KDELR1 expression level and proportions of the immune cells in KDELR1 groups in TCGA. **(A)** Proportions of the 22 types of tumor-infiltrate immune cells in two KDELR1 groups in TCGA. The high-KDELR1-expression group has a higher **(B)** stromal score, **(C)** immune score, and **(D)** ESTIMATE score than the low group in TCGA. The red represents the high-KDELR1-expression group, and the green indicates the low group. ****p < 0.0001. Violin plot showed the ratio differentiation of 22 kinds of immune cells between IDH mutation and wildtype glioma samples using the CIBERSORT algorithm in **(E)** CGGA301 and **(F)** CGGA325 cohorts. *p < 0.1, **p < 0.01, ***p < 0.001.

To explore KDELR1 and immune infiltration in LGG and GBM, KDELR1 was analyzed using the TIMER database. KDELR1 was significantly correlated with dendritic cells in GBM and B cells, CD8+ T cells, CD4+ T cells, macrophages, neutrophils, and dendritic cells in LGG (**Figure 8A**). Moreover, the results showed that B cell, CD8+ T cell, CD4+ T cell, macrophage, neutrophil, and dendritic cell infiltration significantly affected the prognosis (P<0.05) and was correlated with KDELR1 expression in LGG patients but not in GBM patients (**Figure 8B**). We analyzed the proportions of 22 immune cells in the two groups by the CIBERSORT algorithm, which revealed that there were significant differences in the proportions of CD8+ T cells, activated NK cells, monocytes, M1 macrophages, M2 macrophages, and neutrophils between the groups with high- and low-KDELR1-expression levels (**Figure 9A**). The ESTIMATE algorithm was performed to assess the immune levels of glioma patients and showed significant differences (p<0.001) in the immune score, stromal score, and ESTIMATE score between the patients with high and low KDELR1 expression. Specifically, the immune score, stromal score, and ESTIMATE score were all higher in the patients with high KDELR1 expression (**Figures 9B–D**). A similar analysis was conducted in the IDH_mut_ and IDH_wide_ subgroups; the abundances of several immune cells (including CD4 naive T cells, gamma delta T cells, monocytes, M0 macrophages, M1 macrophages, and neutrophils) were different among the two groups (**Figures 9E, F**).

### Functional Enrichment Analysis of KDELR1

**Figure 10:**
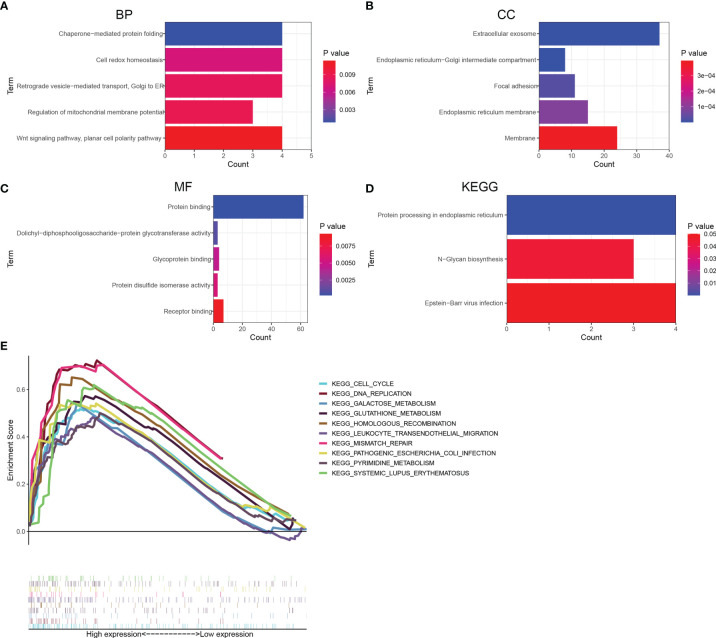
Functional enrichment analyses for top 100 co-expression genes of KDELR1. **(A–C)** Top 5 GO terms of BP, CC, and MF. **(D)** Three KEGG pathways. **(E)** gene set enrichment analysis revealed potential associations between KDELR1 and several metabolism-associated pathways.

To explore the potential function of KDELR1, a total of 100 genes co-expressed with KDELR1 were identified using the GEPIA2 database, and these genes were uploaded to DAVID online. Further Gene ontology (GO) enrichment analysis revealed that these 100 co-expressed genes may be associated with chaperone-mediated protein folding, cell redox homeostasis, the regulation of mitochondrial membrane potential, Wnt signaling, planar cell polarity, extracellular exosome, focal adhesion, endoplasmic reticulum membrane, protein binding, glycoprotein binding, and protein disulfide isomerase activity (**Figures 10A–C**). Interestingly, Kyoto Encyclopedia of Genes and Genomes (KEGG) analysis further suggested that KDELR1 may be involved in protein processing in the endoplasmic reticulum, N-glycan biosynthesis pathway, and Epstein–Barr virus infection (**Figure 10D**). In addition, GSEA indicated that the gene sets specific to the high-KDELR1-expression group were mainly enriched in metabolism-associated pathways (**Figure 10E**).

### Verification of KDELR1 Expression in Glioma Tissues by Immunohistochemistry (IHC)

**Figure 11:**
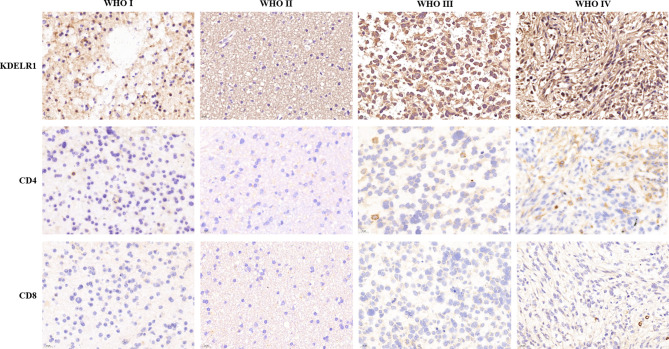
Immunohistochemistry validation of KDELR1 expression and immune infiltration. Columns from left to right are WHO grade I, WHO grade II, WHO grade III, and WHO grade IV, and the rows from top to bottom are the IHC of KDELR1 (1:100, ×20 magnification), CD4 (1:200, ×40 magnification), and CD8 (1:200, ×40 magnification).

To verify the results of the bioinformatics analysis, IHC staining was performed on tissue microarray slides containing the samples of 119 gliomas (5 WHO grade I, 33 WHO grade II, 28 WHO grade III, and 53 WHO grade IV). The level of expression was determined semiquantitatively by the staining index based on staining intensity (SP) and the positive staining percentage (SI). The pathological characteristics and IRS of 119 patients are summarized in **Supplementary Table 1**. The exemplar staining patterns of KDELR1 in the tumors of different grades are shown in **Figure 10**. The expression level of KDELR1 was significantly higher in high-grade glioma than in low-grade glioma tissues (p<0.001), which was consistent with the results of bioinformatics analysis at the RNA level. The relevance between the expression of KDELR1 and immune infiltration was confirmed by the IHC of CD4 and CD8; a positive trend of immune infiltration was consistent with data obtained from the algorithm (**Figure 11**).

## Discussion

Gliomas are the most common primary CNS tumor, accounting for more than 80% of primary brain tumors (30). Among them, GBM is prone to recurrence and has a median survival time of less than 2 years (4). Using bioinformatics analyses, in this study, we found that KDELR1 expression levels were higher in glioma samples than in the corresponding normal tissues. In addition, further evaluation confirmed that KDELR1 was closely related to the clinical features of glioma. These findings suggest that KDELR1 may be a promising biomarker for the precise diagnosis, molecular characteristics, treatment, and prognostic evaluation of gliomas.

It has been established that several clinical features, including age, recurrence, and pathology, are responsible for clinical prognosis in glioma patients (31–33). A previous study also suggested that older glioma patients would have a poorer prognosis than younger patients, which indicates that KDELR1 expression levels might possess an indirect clinical value in prognosis (34). Consistently, our findings also reveal that older patients had higher KDELR1 expression than younger patients. In pathologic analysis, we found that HGGs had higher KDELR1 expression than LGGs. In addition, previous studies (35–37) have demonstrated that mesenchymal-subtype gliomas are associated with poor prognosis, which are confirmed by our finding that mesenchymal -subtype gliomas had a higher expression level of KDELR1 than proneural subtype gliomas. Subsequently, we further explored whether the expression of KDELR1 is related to recurrence. As expected, KDELR1 was more frequently highly expressed in the recurrent group compared with the initial diagnosis group. Taken together, these findings indicate that KDELR1 might act as a novel promising biomarker for the diagnosis, treatment, and prognosis of gliomas.

Recently, with the rapid advances in next-generation technology, research on the biomolecular markers and associated signaling pathways that are involved in the occurrence and development of gliomas has made substantial progress (38). After the IDH mutation status was confirmed to be related to the prognosis of patients with GBMs, a subsequent study reported that the chromosome 1p/19q codeletion status, O6-methylguanine-DNA methyltransferase (MGMT) promoter region genotype, a-thalassemiamental retardation syndrome X (ATRX), and amplification of the epidermal growth factor receptor (EGFR) played more important roles in the prognosis and treatment prediction of gliomas (39, 40). Our study demonstrates that KDELR1 is downregulated in the 1p/19q codeletion group compared with the 1p/19q non-codeletion group. Thus, KDELR1 might be a negative prognostic factor in gliomas from the 1p/19q codeletion perspective. On the other hand, our results indicated that the IDH mutation group had a lower expression level of KDELR1 than the IDH-wildtype group.

In 2010, a study classified GBMs into proneural, neuronal, classical, and mesenchymal subtypes according to the status of Platelet-derived growth factor alpha receptor (PDGFRA), isocitrate dehydrogenase 1 (IDH1), Epidermal growth factor receptor (EGFR), and Neurofibromin type 1 gene (NF1) (8). Additionally, in a study of 107 HGG samples, Phillips et al. divided the samples into three subtypes: proneural, mesenchymal, and proliferation. Proliferation- and mesenchymal-subtype tumors tend to express high levels of genes related to cell proliferation and angiogenesis (9), respectively, and these types often occur in elder (more than 50 years old) patients who have poor prognosis (41). Notably, our research found that KDELR1 is highly expressed in the mesenchymal subtype and expressed at low levels in proneural-subtype gliomas. Based on this evidence, we further inferred, from the molecular classification, that KDELR1 was strongly associated with an unfavorable clinical outcome.

Our previous results have indicated that the increased expression of KDELR1 is strongly related to negative survival factors such as older age, a higher WHO grade, recurrence, IDH wild type, and 1p/19q non-codeletion status; therefore, we further investigated whether the expression of KDELR1 is related to the survival time of patients with gliomas. Survival analyses showed that gliomas with KDELR1 overexpression were associated with shorter OS and PFS times than gliomas with low KDELR1 expression. This finding may provide proof that KDELR1 can be used in predicting clinical prognosis.

We further focused on the relation between KDELR1 and immune infiltration; CD8+ T cells, CD4+ T cells, and macrophages were found with high expression in HGGs along with the overexpression of KDELR1. The IHC expression of CD4 and CD8 in four different grades is consistent with the bioinformation results, and CD8 showed more participants in the tumoral immune microenvironment.

In summary, our findings show that KDELR1 is upregulated in gliomas compared with normal brain tissues and that its expression is significantly associated with clinical features such as the WHO grade, recurrence, molecular classification, IDH mutation, and 1p/19q codeletion status. Moreover, the survival and Cox regression analyses of different datasets suggested that KDELR1 expression in gliomas could be an independent, unfavorable prognostic factor for survival time. On the other hand, KDELR1 expression was associated with immune infiltration (including the infiltration of CD8+ T cells, CD4+ T cells, macrophages, and so on) and microenvironment parameters (including stromal, immune, and ESTIMATE scores) in gliomas. Collectively, these results indicate that KDELR1 could be a promising novel biomarker for molecular classification, immune treatment, and prognostic assessment in glioma.

However, there were some limitations in this study. First, the research on KDELR1 in glioma is still in the early stage, and our research is limited to the bioinformatics database analysis and experimental verification of IHC. Thus, further studies based on surgical samples will be imperative to perform *in vitro* or *in vivo* assays, validating these findings, and as a biomarker, diagnostic tests will be conducted in future research. Second, bioinformation data collected from the online database were established and categorized based on the 2016 WHO CNS classification system. Since the 2021 WHO classification system has been published, a prospective study is underway to collect samples and improve this work.

## Data Availability Statement

The raw data supporting the conclusions of this article will be made available by the authors, without undue reservation.

## Ethics Statement

The studies involving human participants were reviewed and approved by the Ethics Committee of Shanghai OutDo Biotech Co., LTD. and the Ethics Committee of Huashan Hospital. The patients/participants provided their written informed consent to participate in this study.

## Author Contributions

YY, BY, and ZQ performed the research. ZH conducted the IHC staining. JC designed the research. YY and BY wrote the article. JS revised it. All authors contributed to the article and approved the submitted version.

## Funding

This work was supported by the Shanghai Municipal Science and Technology Major Project (No. 2018SHZDZX01) and ZJLab, the Shanghai Rising-Star Program (No. 18QA1400900), and Shanghai Hospital Development Center (No. SHDC2020CR3073B).

## Conflict of Interest

The authors declare that the research was conducted in the absence of any commercial or financial relationships that could be construed as a potential conflict of interest.

## Publisher’s Note

All claims expressed in this article are solely those of the authors and do not necessarily represent those of their affiliated organizations, or those of the publisher, the editors and the reviewers. Any product that may be evaluated in this article, or claim that may be made by its manufacturer, is not guaranteed or endorsed by the publisher.
