# Supplementary material for: KDELR1 Is an Independent Prognostic Predictor and Correlates With Immunity in Glioma
Source: Front Oncol. 2022 Jun 23;12:783721. doi: 10.3389/fonc.2022.783721 (PMC9263977; doi:10.3389/fonc.2022.783721)
Supplement: Supplementary file 1 [file Table_1.docx]

Sup Table 1

Pathological characteristics and IRS of 119 patients of glioma tissue microarray

| Tumor Grade | Immunoreactivity Scores | | | Total |
| --- | --- | --- | --- | --- |
|  | +(2-3) | ++(4-5) | +++(6-7) |  |
| I | 5 | 0 | 0 | 5 |
| II | 22 | 11 | 0 | 33 |
| III | 3 | 17 | 8 | 28 |
| IV | 0 | 6 | 47 | 53 |
| total | 30 | 34 | 55 | 119 |
